# Superhydrophobic Nanocomposite Polysulfone Fibrous Membranes Incorporated with Phenyltriethoxysilane-Modified Silica for Oil–Water Separation

**DOI:** 10.1021/acsomega.5c05708

**Published:** 2025-12-15

**Authors:** Safiye Gözde Palamutcu, Zuleyha Sarac, Cigdem Tasdelen-Yucedag

**Affiliations:** Department of Chemical Engineering, 52962 Gebze Technical University, Gebze, Kocaeli 41400, Turkey

## Abstract

Oily wastewater originating from industrial activities and domestic effluents has been one of the major sources of pollution, leading to contamination of clean water supplies and jeopardizing aquatic ecosystems. Water pollution accompanied by water scarcity manifests the compelling need for advanced next-generation membrane-based treatment strategies. In this study, high flux, robust, durable, and superhydrophobic nanocomposite fibrous membranes including silica nanoparticles were produced via electro-blow spinning (EBS) of polysulfone (PSU). To enhance water repellency, the surface of nanoparticles was functionalized with phenyltriethoxysilane (PTES). The amount of disperse phase was optimized by the addition of PTES-modified silica (SPTES) nanoparticles into the polymer spinning solution at changing ratios between 1 and 10 wt %. The best membrane performance was obtained with the membrane specimen including 5 wt % SPTES (PSU@SPTES-5). This SPTES ratio stimulated the dense distribution of silica nanoparticles, resulting in distinct surface roughness. Due to the incorporation of SPTES, the water contact angle (WCA) of pristine PSU membrane was enhanced from hydrophobic (129.9°) range to superhydrophobic (154.3°) range. The PSU@SPTES-5 membrane sample exhibited excellent performance in both strength and elongation, maintaining durability while preserving flexibility. Furthermore, its initial decomposition temperature increased from 437 to 461 °C, while char yield shifted from 12.2 to 30.9% relative to PSU. The separation efficiencies of PSU@SPTES-5 were measured as 99.4%, 96.6%, 99.9%, and 86.5% for diesel, CCl_4_, petroleum spirit, and sunflower oil (SFO), respectively. After 20 consecutive separation cycles, PSU@SPTES-5 retained a separation efficiency above 97.2%. The efficiency of 86.5% at gravity-driven separation increased to 98.6% when the pressure-driven system was employed for SFO. Upon testing against diesel- and SFO-in-water emulsions (99:1 = O/W (w/w)), PSU@SPTES-5 sample showed fluxes of 1203 L/m^2^ h and 63L/m^2^ h, with corresponding separation efficiencies of 99.3% and 97.2%, respectively. When evaluated under harsh conditions, even after 24 h exposure to 2 M HCl, 2 M NaOH, and UV irradiation, the membrane retained its separation efficiency above 97% in all cases after 20 cycles.

## Graphical abstract

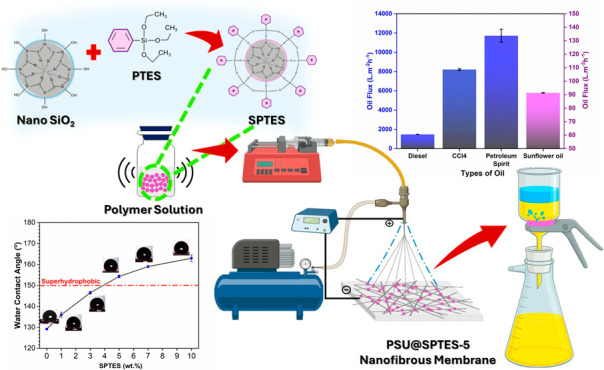

## 1 Introduction

By 2030, nearly 3.8 billion people are projected to face water scarcity, while more than 1.2 billion already lack access to clean drinking water.^1,2^ This escalating water crisis underscores the urgent need for advanced water purification technologies to address growing environmental and industrial pressures.^2–4^ Industrial activities such as textile production, food processing, leather manufacturing, metalworking, petroleum refining, gas extraction, and mining generate substantial volumes of oily wastewater. The release of untreated oily wastewater and incidental oil spills pose significant environmental and health risks, contributing to global pollution. According to the Environmental Protection Agency and the Environmental Defense Fund, as clean water resources continue to deplete at an alarming rate, the removal of oil from wastewater prior to environmental discharge has emerged as a critical concern for sustainable water management.^5,6^

Conventional methods including gravity separation, coagulation–flocculation, solvent extraction, adsorption, and precipitation are commonly utilized for the treatment of oily wastewater.^7–9^ Nevertheless, limitations such as low oil absorption capacity, poor recyclability, and the risk of secondary pollution serve as driving forces leading to the development of more advanced and efficient separation strategies. Hence, membrane separation technologies have been acknowledged as next-generation approaches with their superior separation performance, reduced energy requirements, and ease of integration into treatment systems.^10^ Among various membrane-based technologies, nanofiber membranes demonstrate enhanced separation performance for oil–water separation, due to high specific surface area, fine pore structure, and tunable surface functionalities.^9,11^

Electrospinning has been a well-established and versatile technique capable of producing continuous fibers with diameters ranging from micrometers to a few nanometers, which renders possible the fabrication of nanofiber membranes with high porosity, tunable pore size, and controllable morphology. However, certain constraints such as low production rates and the need for high-voltage operation confine its scalability.^11–14^ To overcome these challenges, the electro-blow spinning (EBS) technique has been developed, which utilizes both electrostatic and high-velocity air forces to produce nanofibers with smaller diameters, narrower fiber distribution, and acceptable bead/droplet density. Through the synergistic effects of simultaneously applied electric fields and high-velocity airflow, EBS not only enhances productivity but also minimizes structural defects, resulting in more uniform and reliable nanofiber formation.^13^

Nanofiber production technology paves the way for the exploitation of various polymers and their combinations in the production of membranes targeting oil–water separation processes. The most frequently employed polymers include polyvinylidene fluoride (PVDF),^11^ polyvinylpyrrolidone (PVP),^15^ polysulfone (PSU),^13^ polyethylene terephthalate (PET),^16^ and polyacrylonitrile (PAN),^17^ due to their favorable features such as chemical and mechanical stability, and engineered surface wettability. Among them, PSU is particularly notable for its outstanding thermal, chemical, and mechanical stability, processability, and excellent hydrolytic resistance.^13^

Tailored surface wettability, which should be carefully considered in membrane design, constitutes a key parameter in achieving efficient oil–water separation. In particular, nanofiber membranes offering superhydrophobic properties have been identified as highly effective solutions for oil–water separation by virtue of their superior water repellency and selective oil permeability.^11,12,14,18^ Recent studies have concentrated on improving the hydrophobicity and separation efficiency of nanofiber membranes through the incorporation of functional nanoparticles, aiming to enhance their performance in oil–water separation applications.^8^ Nanoparticles such as titanium dioxide (TiO_2_), silicon dioxide (SiO_2_), graphene oxide (GO), and iron­(II,III) oxide (Fe_3_O_4_) have been incorporated into nanofiber structures to enhance the hydrophobicity.^11,19^ The nanoscale size of these particles enables the formation of nanoscale roughness on the membrane surface, which plays a key role in improving water-repellent properties. Obaid et al. prepared PSU-based electrospun nanofiber membranes containing SiO_2_ nanoparticles and GO nanosheets by using electrospinning for oil–water separation applications. The presence of SiO_2_ nanoparticles in the membrane structure induced improvement in both mechanical properties and water contact angle (WCA), and caused a dramatic increase in flux. Meanwhile, the incorporation of GO nanosheets deteriorated the mechanical strength and led to a slight increase in flux in comparison to the pristine PSU membrane.^20^ Similarly, the addition of 3 wt % of SiO_2_ into PVDF nanofibers enhanced the WCA from 138.5° to 150.0° and gave a high filtration efficiency (∼99%) with excellent reusability.^7^

Furthermore, the large surface area of nanoparticles facilitates chemical functionalization which promotes stronger interaction between low-surface-energy materials and nanoparticles, thereby contributing to the formation of hydrophobic surfaces with enhanced oil–water separation performance.^11^ Building upon that, the surface modification can be applied to the nanoparticles by using organosilanization agents through sol–gel to intensify the hydrophobicity.^21^ It was reported that after the modification of SiO_2_ nanoparticles by using organosilanes such as *n*-octyltriethoxysilane, vinyltrimethoxysilane, and vinyltriethoxysilane, the PVDF-based nanofiber membranes demonstrated an increase in WCA from 149.8° to 160.1° as the additive amount increased from 0.5% to 5%.^22^ The surface of SiO_2_ nanoparticles was treated with perfluorooctyltriethoxysilane (PFOTES) by Yang et al. in order to improve the surface roughness and the hydrophobicity.^9^ The PVDF-based nanofibrous mats which were fabricated with these PFOTES-modified silica nanoparticles via electrospinning attained a superior hydrophobicity and high separation efficiency in emulsified oil–water systems.^9^ These studies have revealed that the incorporation of modified nanoparticles into the nanofibrous membranes could augment superhydrophobicity and separation efficiency, transforming them into promising candidates for oil–water separation applications.

In this study, the surface of nanoparticles was modified with phenyltriethoxysilane (PTES) organosilane coupling agent through sol–gel reaction to attain superhydrophobic nanoparticles. High flux, robust, and durable nanocomposite fibrous membranes were fabricated with PSU using EBS method combined with SPTES nanoparticles for oil–water separation applications. In order to optimize the amount of incorporated nanoparticles, SPTES was added into the polymer solution at varying ratios (1–10 wt %). To the best of our knowledge, this is the first study introducing the SPTES-modified PSU nanocomposite fibrous membranes for oil–water separation purposes. The prepared nanocomposite fibrous membranes were comprehensively characterized in terms of fiber morphology, surface wettability, mechanical and thermal properties, oil–water separation performance, and stability under harsh conditions.

## 2 Experimental Section

### 2.1 Materials

Hydrophilic fumed silica (Aerosil 380) was purchased from Germany, Evonik Industries. Phenyltriethoxysilane (PTES, 98%), ammonium hydroxide (NH_4_OH, 25–30%), and ethyl alcohol (C_2_H_5_OH, ≥99.9%) were purchased from Sigma-Aldrich. Polysulfone (PSU-UDEL-P1700 LCD, *M*_W_ = 65,000–75,000 Da) granules were kindly provided by SOLVAY, Germany. *N*,*N*-Dimethylformamide (DMF, ≥99%) was obtained from Carlo Erba, while isopropyl alcohol (IPA, ≥99.5%) was purchased from Merck. Diesel oil was purchased from a local gas station of the city. Carbon tetrachloride (CCL_4_, 95%) and petroleum spirit (≥90%) were obtained from BDH Chemicals Ltd. Sunflower oil (SFO) was obtained from the local supermarket in the city. Sudan IV and methylene blue were purchased from Sigma-Aldrich, were used to color oil and water phases, respectively.

### 2.2 Preparation of PTES-Modified Silica (SPTES) Nanoparticles

For the preparation of hydrophobic silica nanoparticles, a procedure was adapted from the literature for the sol–gel reaction.^23–25^ Surface modification of Aerosil 380 nanoparticles was carried out using PTES as a silane coupling agent. The PTES/SiO_2_ weight ratio (R) was varied in the range of 0.12–1.2 by adjusting the amount of PTES while keeping the SiO_2_ content constant.

First, a dispersion solution was prepared by dissolving 4 mL of NH_4_OH (used as a catalyst) in 16 mL of H_2_O. Then, Aerosil 380 nanoparticles were added and dispersed in the resulting solution. The well dispersed silica suspension was added to the PTES-ethanol solution, which had been prepared previously. The reaction mixture was continuously stirred at 400 rpm and 40 °C for 24 h. After the reaction was completed, the resulting solution was centrifuged at 4500 rpm and washed with ethanol, and subjected to centrifugation again. This cycle was employed 3 times to ensure thorough purification. The modified nanoparticles were subsequently dried at 60 °C under vacuum for 24 h.

### 2.3 Fabrication of Nanofibrous Membranes Containing SPTES (PSU@SPTES-X)

The suspensions containing PTES-modified silica at different concentrations (1, 3, 5, 7, and 10 wt %) were prepared by dispersing the SPTES nanoparticles in DMF using magnetic stirrer at 400 rpm for 1 h at room temperature. Next, the mixtures were subjected to ultrasonication in a bath for 20 min to disperse the SPTES nanoparticles homogeneously. Subsequently, PSU was dissolved in DMF at a concentration of 13 wt %, which had been optimized in our previous study.^13^ All prepared solutions were then subjected to mechanical stirring at room temperature for 24 h. Nanocomposite fibrous membranes were produced by the EBS method according to the optimized conditions reported in our earlier study.^13^ The optimum values of air pressure and voltage were 3 bar and 7.5 kV, respectively. The resulting membranes were labeled as PSU@SPTES-X, where X denotes the amount of SPTES nanoparticles incorporated into the membranes.

### 2.4 Characterization

Fourier transform infrared (FTIR) spectroscopy was employed to investigate the chemical changes in the structure of pristine hydrophilic silica after the modification with PTES. The FTIR spectra of nanoparticles were recorded using a Bruker Tensor 37 spectrometer equipped with an attenuated total reflectance (ATR) accessory. Spectra were obtained with 32 number of scans in the wavenumber range of 4000–500 cm^–1^. Bruker’s OPUS software was used to determine peak positions, apply baseline corrections, and process the spectra. The mean particle diameter of SPTES nanoparticles was measured by dynamic light scattering (DLS) using a Zetasizer Nano-ZS90 system (Malvern Inc.) Scanning electron microscopy (SEM) analysis was performed using a Philips XL30 SFEG to examine the morphology of PSU@SPTES-X nanocomposite fibrous membranes and the distribution of SPTES nanoparticles on the nanofibers. The average fiber diameter (AFD) values and fiber diameter distribution of PSU@SPTES-X membranes were analyzed from SEM images through image analysis software (ImageJ). AFD values and standard deviation of the nanofiber samples were calculated through measurements made using at least 100 different fibers.

The porosity of PSU@SPTES-X nanocomposite fibrous membranes was determined by a gravimetric method based on weighing the liquid taken up by the membrane pores. IPA was used as the wetting liquid due to the hydrophobic nature of the membrane. The porosity (%) values of the membranes were calculated using the equation in our previous study.^13^ The wettability behavior of SPTES nanoparticles and PSU@SPTES-X nanocomposite fibrous membranes was characterized using a contact angle meter (KSV CAM 200) with a 5 μL water droplet. The average water contact angles (WCAs) of the samples were calculated using measurements obtained from three different regions of the samples. The mechanical properties of the membranes were measured using a Devotrans DVT BP D NN tensile testing machine (Turkey) following the ISO 9073-3:1989 standard. The samples (50 × 200 mm) were tested at a tensile speed of 5 mm/min. The detailed methodology for mechanical testing was adopted from our previous work.^13^ Thermogravimetric analysis (TGA) was performed via using a Mettler Toledo TGA/SDTA 851 instrument equipped with STAR software, with a heating rate of 10 °C/min under a nitrogen flow of 50 mL/min, from room temperature to 900 °C.

### 2.5 Separation Performance Test

For the oil–water separation performance test, the obtained PSU@SPTES-X nanocomposite fibrous membranes were sandwiched between the glass funnel and conical flask of filtration apparatus. The oil–water systems were prepared by mixing water with different types of oils at a 1:1 volume ratio. Diesel, CCl_4_, petroleum spirit, and SFO were employed as the oil phase. After the membrane specimen was mounted into the filtration apparatus, the oil–water mixture was poured from the top of the glass funnel and the duration of oil permeation through the membrane driven naturally by gravity was recorded. The measured duration corresponds to the interval between the onset of separation and the point at which oil permeation through the membrane ceased. The permeate flux (*J*) was calculated according to Fick’s first law of diffusion:^15^

1 $$J=\frac{V}{A\times t}$$

where *J* is the permeation flux (L/m^2^ h), *V* is the volume of permeated oil (L), and *A* and *t* are effective membrane filtration area (m^2^) and permeation time (*h*), respectively. The separation efficiency was calculated using the weighed amounts of oil before and after separation, according to the following equation:^15,26^

2 $$E_{1}\left(\%\right)=\frac{m}{m_{0}}\times 100$$

where *m*_0_ and *m* represent the weights of oil feed and permeate, respectively.

WCA, Young’s modulus, flux, and separation efficiency measurements were conducted in triplicate (*n* = 3). Statistical analysis was carried out using Tukey’s multiple comparison test, with significant differences indicated by distinct letters on the figures. The values reported in the text represent mean values, while standard deviations are provided in the corresponding figures.

The durability of the membranes was assessed by repeating the gravity-induced oil–water mixture separation for 20 cycles. In addition, to elucidate the membrane’s performance under dynamic operational conditions and demonstrate its potential for practical applications, an alternative pressure-driven test setup was employed.^27,28^ The plunger of horizontally positioned syringe on the syringe pump, including the SFO-water mixture, was actuated at a constant flow rate of 2 mL/min, enabling a faster and continuous separation.^27^ When the SFO-water mixture was forced to flow through this pressure-driven system, SFO could easily pass through due to the superoleophilicity of the membrane, whereas the water phase failed to permeate because of superhydrophobicity.

Water-in-oil (W/O) emulsions were freshly prepared by introducing deionized water (1 wt %) into diesel using 0.1 wt % Span 80 as the surfactant, followed by vigorous magnetic stirring at 1200 rpm under ambient temperature until a stable, milky white dispersion was obtained. The same procedure was applied for SFO to prepare the corresponding W/O emulsion.

The separation efficiency (*E*_2_) was calculated using equation:^29^

3 $$E_{2}\;\left(\%\right)=\left(1-\frac{C_{P}}{C_{0}}\right)\times 100$$

Here, *C*_0_ and *C*_P_ are the water concentrations of the feed emulsion and permeate, respectively. Water content measurements were performed using a Karl Fischer moisture titrator (Metrohm KF 915 Ti-Touch, Switzerland). Optical microscopy images of the emulsions were obtained using an optical microscope (Nikon Eclipse LV 100D/Symantec 3D) by placing a drop of the sample onto a transparent glass slide. All experiments in this study were repeated three times.

### 2.6 Stability Tests

The chemical stability of the PSU@SPTES-5 was tested by 24 h immersion in 2 M HCl and 2 M NaOH solutions.^29^ After immersion, the membranes were rinsed with deionized water, dried, and then tested for oil–water separation performance with diesel over 20 repeated cycles, recording both flux and separation efficiency. In addition, changes in surface wettability were examined by measuring the WCA values after different immersion times in acidic and alkaline media. To further assess environmental durability, the membranes were also exposed to direct sunlight for 24 h, after which their separation performance was tested again using diesel under the same conditions. These procedures were designed to simulate harsh operational environments and to provide a clearer understanding of the membranes’ chemical stability and long-term performance.

## 3 Results and Discussion

Herein, high flux, mechanically robust, and durable superhydrophobic nanocomposite fibrous membranes (PSU@SPTES-X) were developed through the incorporation of PTES-modified nanoparticles into PSU nanofibers fabricated by EBS technique for oil–water separation applications, as illustrated in Figure 1. A series of electro-blown spun nanocomposite fibrous membranes were produced by adding varying amounts of SPTES. The resulting PSU@SPTES-X membranes were tested for oil–water separation using two distinct setups: gravity-driven separation and pressure-driven separation conducted with different types of oils.

**Figure 1:**
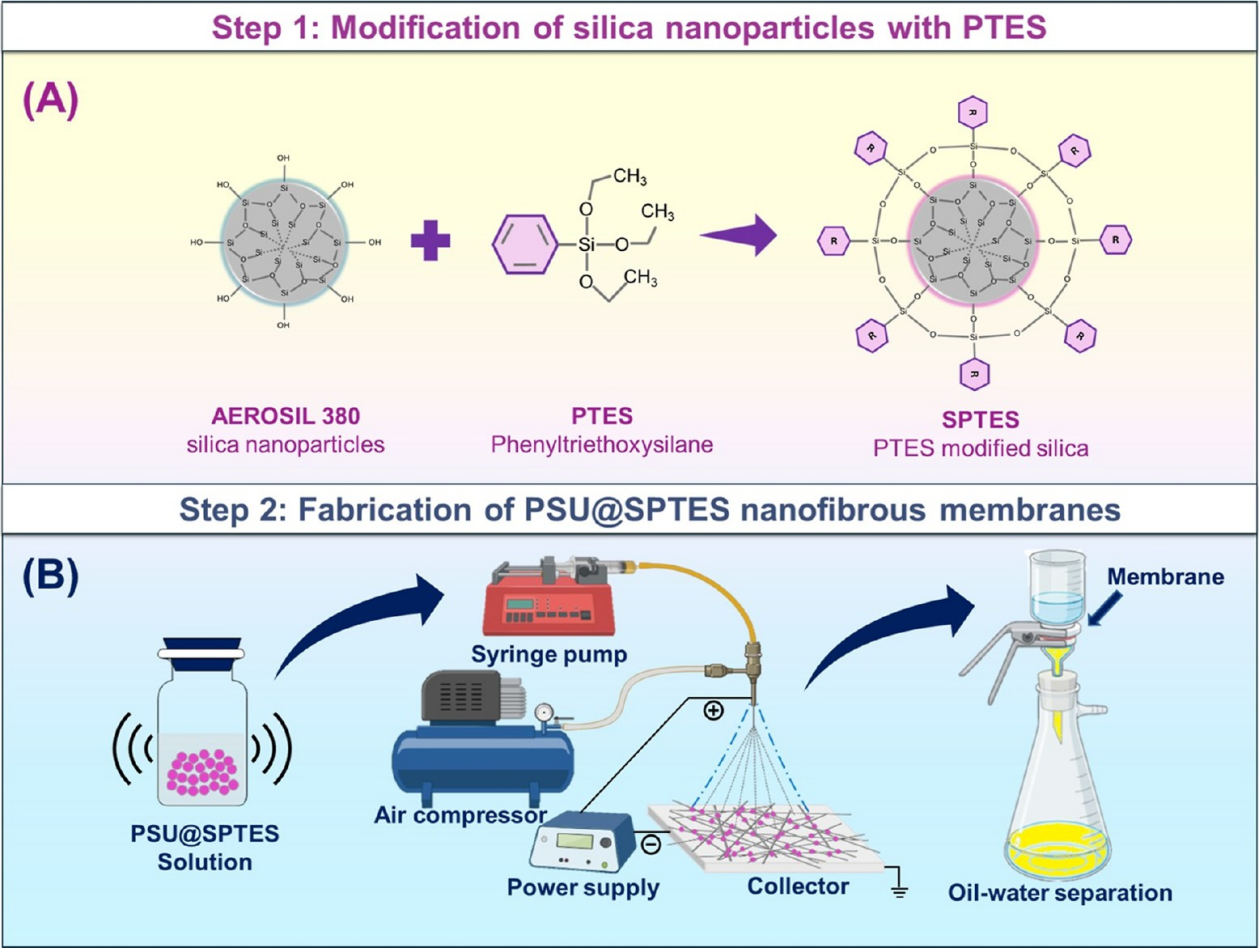
Schematic representation of fabrication of; (A) surface modification of nanoparticles, and (B) fabrication of PSU@SPTES-X nanocomposite fibrous membranes via EBS.

### 3.1 Optimization and Characterization of Surface Modified Nanoparticles

The surface modification reaction of nanoparticles with PTES was optimized to achieve superhydrophobic characteristics. For this purpose, a series of reactions were carried out at different PTES/SiO_2_ ratios (*R*), with the experimental details provided in the previous section.

FTIR spectra were recorded to confirm whether the nanoparticles were successfully coated with PTES organosilane agent. Figure 2 shows the FTIR spectra of the silica samples subjected to modification through different reaction conditions (*R* = 0.12, 0.2, 0.6, and 1.2).

**Figure 2:**
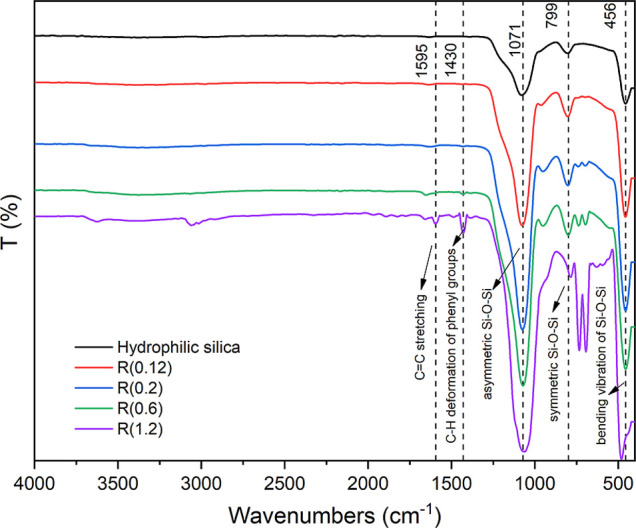
FTIR spectra of unmodified silica and PTES-modified silica samples (*R* values represent different modification ratios).

In the spectrum of the specimen which prepared by using PTES/SiO_2_ ratio R(0.6) the stretching vibration of C–H bonds in phenyl groups were observed at 2997 cm^–1^, while the in-plane stretching of C=C bonds appeared at 1595 cm^–1^. The simultaneous presence of C=C stretching and in-plane C–H deformation of phenyl groups was detected at 1430.44 cm^–1^. The Si–C stretching, confirming the attachment of the phenyl group to the silicon atom, was observed at 737.30 cm^–1^. The peaks at 1071.34 cm^–1^, 799.59 cm^–1^, and 456.25 cm^–1^ correspond to the asymmetric, symmetric, and bending vibrations of the Si–O–Si bonds in silica, respectively. The band at 951.20 cm^–1^ is attributed to the in-plane stretching vibrations of Si–OH groups, while the band at 1653 cm^–1^ represents the deformation vibration of adsorbed water molecules. All of these results are consistent with the literature,^23,30^ confirming the structure shown in Figure 1A.

In the FTIR spectrum of pristine SiO_2_ nanoparticles, the broad −OH band observed in the range of 3200–3600 cm^–1^ corresponds to silanol (−Si–OH) groups on the surface of silica.^31–33^ During the modification process, according to the sol–gel reaction mechanism, the intensity of this band was anticipated to decrease as the −Si–OH groups of hydrolyzed PTES reacted with the surface hydroxyl groups of pristine silica. However, when the spectrum of pristine silica was compared with that of PTES-modified silica, this effect was not clearly observed. This might be explained by the relatively low intensity of hydroxyls on the surface of pristine silica compared to other peaks in the spectrum. Moreover, when spectra of the different reactions conducted at different PTES/SiO_2_ ratios were elucidated, a progressive increase was observed in the intensity of the −OH peak with increasing PTES amount. This trend could be because of the detection of the hydroxyl groups of hydrolyzed but unreacted PTES molecules. In brief, intensified peak between 3200 and 3600 cm^–1^ is probably associated with the presence of hydrolyzed PTES molecules that neither reacted with nanoparticles nor underwent self-condensation due to steric hindrances.^23,30^

Next, WCA measurements were executed to further confirm the hydrophobic modification of silica surface via sol–gel reaction, as indicated in Figure 3A,B.

**Figure 3:**
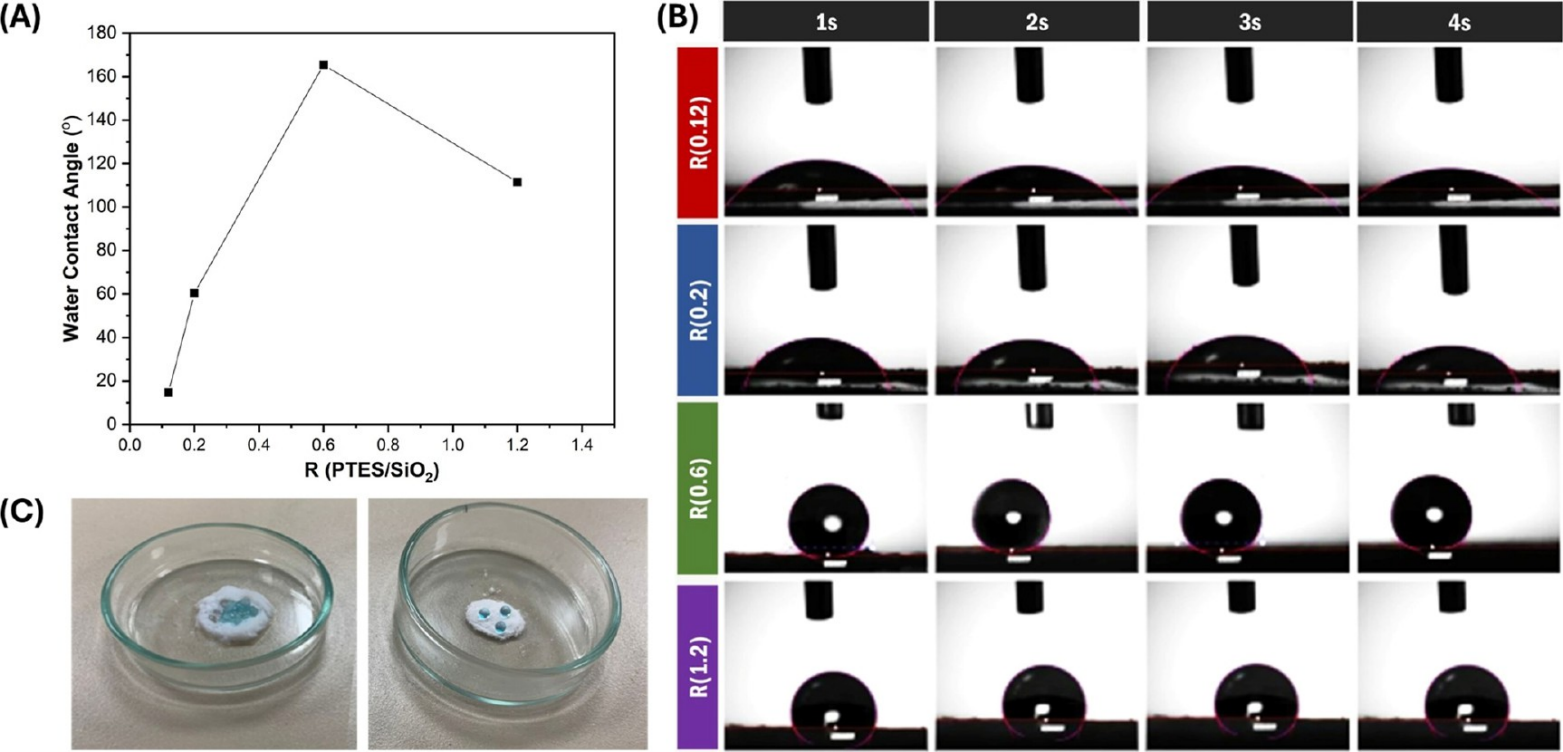
(A) Effect of R­(PTES/SiO_2_) ratiosof silica samples; (B) time-dependent wetting behavior of nanoparticles with different modification ratios; (C) water droplet behavior on unmodified and PTES-modified silica nanoparticles.

The data show that R(0.6) exhibited superhydrophobic properties with a WCA of 165.5°. In addition, the WCA of R(1.2) was measured as 111.5°, which is lower than the superhydrophobicity threshold. This variation may stem from the side interactions due to excess PTES and insufficient surface modification. These results confirm that the optimal reaction conditions were achieved in the sample with R(0.6). A visual comparison of the PTES-modified sample before and after modification in the reaction R(0.6) is seen Figure 3C (Video S1). In addition, DLS was adopted to examine hydrodynamic diameter of the nanoparticles. After the surface modification with PTES, the particle size of pristine silica increased from 147.9 to 170.3 nm (PDI:0.67). This predicted increment in the shear plane of modified nanoparticles arises from the change in surface charge caused by phenyl groups of PTES.

### 3.2 Morphology of PSU@SPTES-X Nanocomposite Fibrous Membranes

The morphologies and AFDs of PSU@SPTES-X nanofiber membranes, where X represents the SPTES nanoparticles content (1, 3, 5, 7, and 10 wt %), were analyzed at 100 different randomly selected points using ImageJ software on SEM images as shown in Figure 4. In the PSU@SPTES-1 sample, SPTES particles were sparsely distributed on the surface of nanofibers, whereas their presence was more clearly observed in PSU@SPTES-3 sample. The SEM image (Figure 4C) of the membrane with 5 wt % SPTES revealed that the microspheres were more densely distributed on the nanofibers, indicating a robust integration of SPTES nanoparticles within the nanofiber matrix and resulting in distinct surface roughness. Furthermore, SPTES nanoparticles appeared to be fully embedded within the PSU nanofibers, leading to the development of numerous surface protrusions.^34^

**Figure 4:**
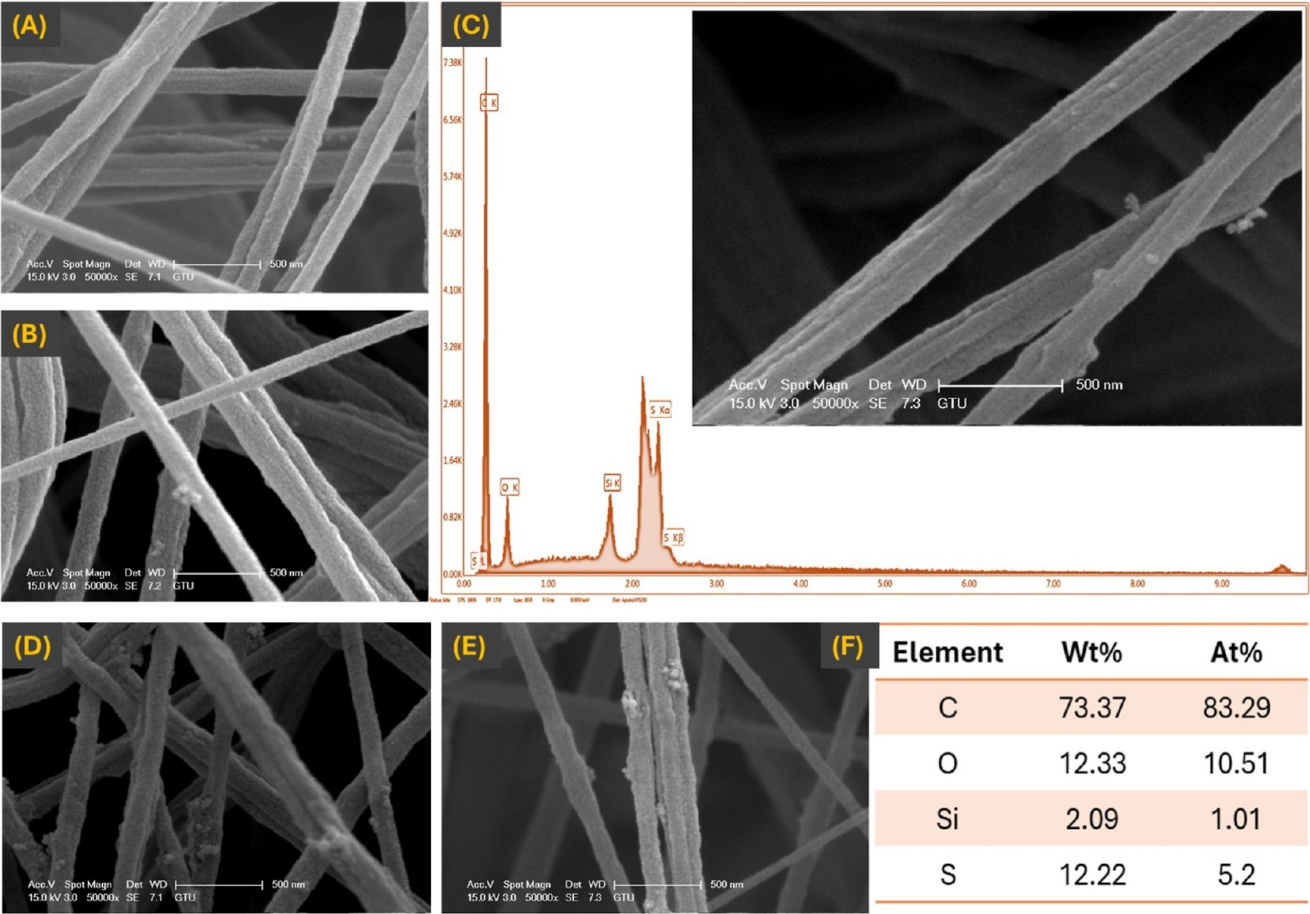
SEM images of PSU nanofibers produced with EBS; (A) PSU@SPTES-1, (B) PSU@SPTES-3, (C) PSU@SPTES-5, (D) PSU@SPTES-7, (E) PSU@SPTES-10, and (F) EDS analysis of PSU@SPTES-5.

However, at an SPTES weight percentage of 7 wt %, the nanofibers exhibited improper stretching, and a further increase in SPTES content to 10 wt % led to the formation of a significant number of beads on the nanofiber surfaces. These observations indicate that the optimal SPTES concentration is 5 wt %, as it provided uniform particle distribution and preserved the structural integrity of the nanofibers. Therefore, further characterization using SEM–EDX was conducted exclusively for the PSU@SPTES-5 membrane to determine its elemental composition. As shown in Figure 4F, the elemental composition of the PSU@SPTES-5 sample was C (73.37%), S (12.22%), Si (2.09%), and O (12.33%), whereas the corresponding atomic percentages were identified as C (83.29%), S (5.2%), Si (1.01%), and O (10.51%), respectively. Thus, the presence of silicon (Si) atoms was confirmed through the EDX analysis.

Next, Table 1 and Figure 5 represent the fiber diameters and their distributions of the membrane samples, respectively. As seen in Table 1, the AFD for the neat PSU nanofibers was calculated as 107 ± 20 nm. Upon the incorporation of 1 and 3 wt % SPTES, the AFD increased to 147 ± 38 nm and 150 ± 48 nm, respectively, indicating that even low levels of SPTES influence fiber thickening. As the SPTES ratio increases to 5 wt %, the fiber diameter consistently increases to 152 ± 43 nm. This growth can be attributed to the influence of nanoparticles on the physical and electrical properties of the solution during the electro-blow spinning process. However, at higher SPTES ratios of 7 and 10 wt %, a reduction in AFD was observed. Additionally, excessive nanoparticle content may disrupt the homogeneity of the spinning solution, leading to instability in the fiber jet and a reduction in fiber diameter.^35,36^ As depicted in Figure 5, it is evident that the morphology of the electro-blown spun fibers was significantly influenced by the addition of SPTES nanoparticles, exhibiting a broad distribution across all samples.

**Table 1:** Porosity and AFDs of Membrane Samples with Different SPTES Ratios

sample	AFD (nm)	porosity (%)
PSU	107 ± 20	88
PSU@SPTES-1	147 ± 38	86
PSU@SPTES-3	150 ± 48	86
PSU@SPTES-5	152 ± 43	88
PSU@SPTES-7	134 ± 35	86
PSU@SPTES-10	122 ± 35	86

**Figure 5:**
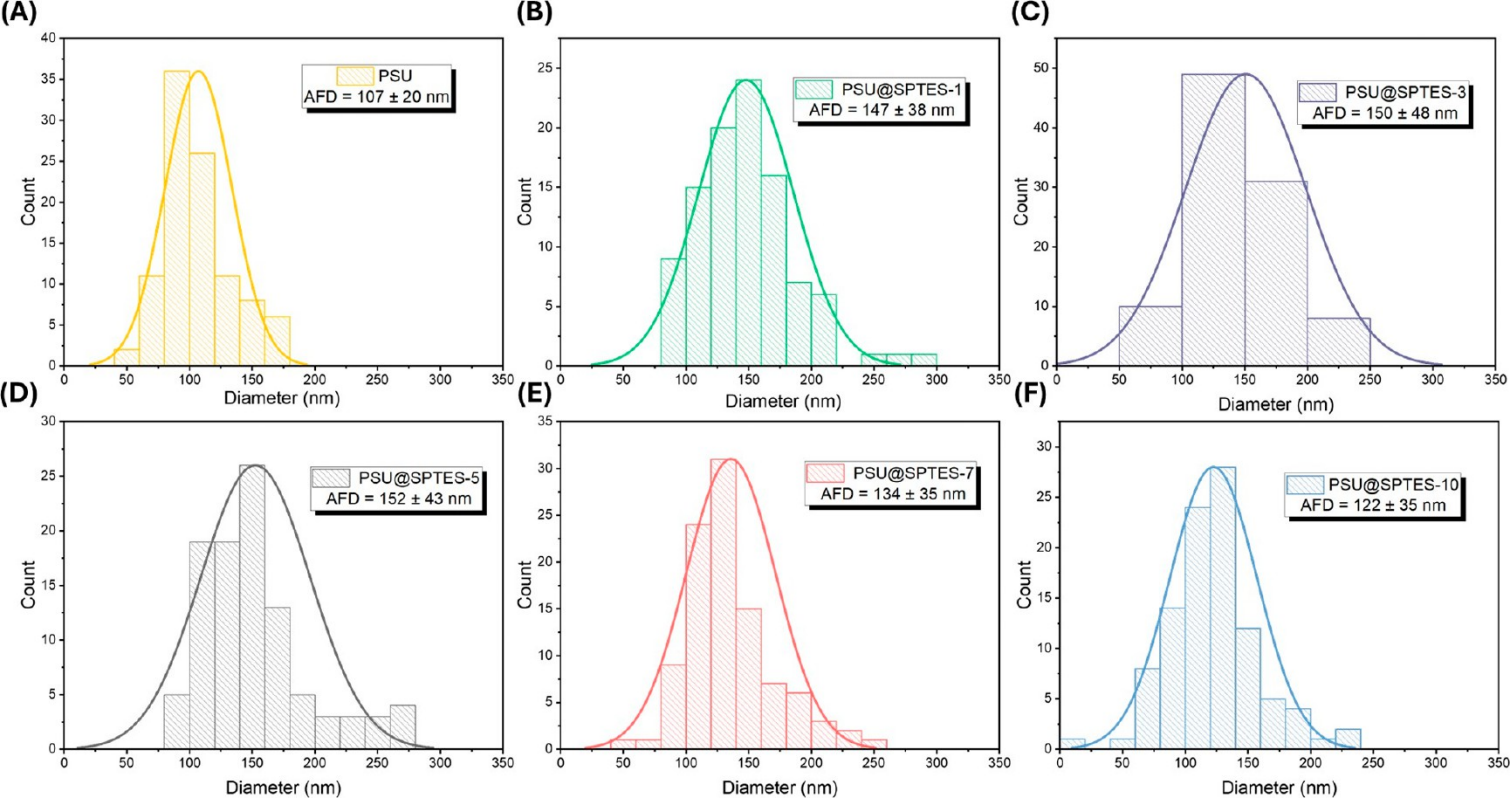
Fiber diameter distributions of (A) PSU, (B) PSU@SPTES-1, (C) PSU@SPTES-3, (D) PSU@SPTES-5, (E) PSU@SPTES-7, and (F) PSU@SPTES-10.

Table 1 also presents the porosities of membrane samples with different SPTES weight percentages. The porosity of the neat PSU membrane was measured as 88%. When SPTES nanoparticles were integrated, the porosity values ranged between 86% and 88%, exhibiting no systematic variation in either direction.

These morphological modifications, particularly the pronounced surface protrusions and uniform nanoparticle embedding observed at 5 wt % SPTES (AFD: 152 ± 43 nm; porosity: 87%), conferred a hierarchical, multiscale roughness along with a porous topology. At the molecular level, such complex roughness enhances surface hydrophobicity by augmenting air entrapment beneath water droplets. This mechanism stabilizes the water–air–solid interface and prevents complete wetting.^37^ Moreover, creating a low-surface-energy chemistry in combination with the multiscale roughness contributes to a superhydrophobic character that strongly repels water while allowing oil penetration, as demonstrated in recent reviews on oil–water separation membranes.^38^ In contrast, the bead formation and irregular fiber stretching observed at 7–10 wt % SPTES with reduced AFD disrupted the uniformity of the fibrous network. The loss of structural regularity and decreased porosity hindered oil flux by restricting transport pathways. This suggests that excessive SPTES loading limits permeation due to morphological densification, ultimately reducing the overall permeability of the membrane.^39,40^

### 3.3 Surface Wettability

Surface wettability is related to both surface chemistry and roughness, each of which plays a crucial role in determining the hydrophobic or hydrophilic nature of a material.^41^ The WCAs of PSU membranes incorporated with SPTES nanoparticles at different weight ratios (SPTES/PSU, wt %) were measured to examine the wettability behavior. Figure 6 presents the WCA values of the membranes corresponding to different SPTES weight ratios.

**Figure 6:**
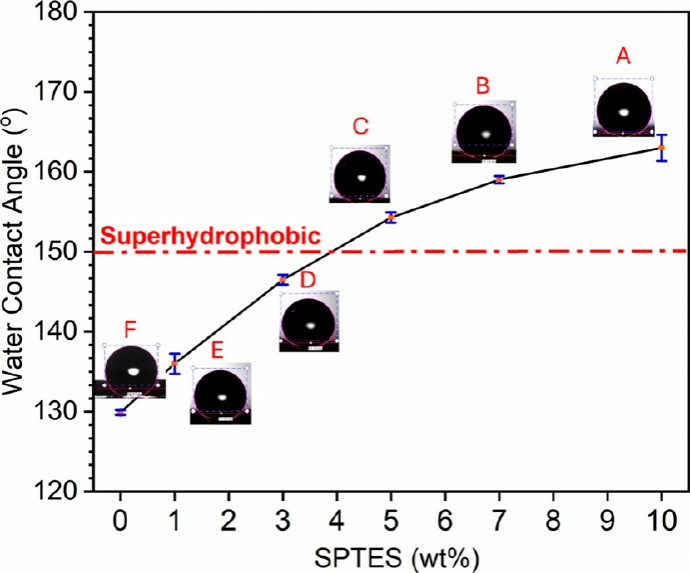
WCAs of pristine PSU nanofibrous membrane and its composites containing different SPTES weight percentages.

The results demonstrated that incorporation of SPTES nanoparticles notably enhanced the water-repellent properties of the PSU@SPTES-X membranes. As the SPTES content of membranes increased from 0 to 10 wt %, the WCA value exhibited a significant rise from 129.9° to 163.0°, pointing out a substantial improvement in surface hydrophobicity. In particular, when the SPTES content reached 5 wt %, the WCA increased to 154.3°, signifying a transition from hydrophobic to superhydrophobic behavior. Although the additive ratios of 7 and 10 wt % induced the WCA to attain superhydrophobic values, the samples with these ratios were found to be insufficient in terms of morphological and mechanical performance, which is discussed in detail in the subsequent sections. All membranes exhibited statistically significant differences in their WCA values (*p* < 0.05), which clearly demonstrates the pronounced effect of SPTES content on surface wettability.

### 3.4 Mechanical Stability

The mechanical performance of the fabricated nanocomposite fibrous membranes was evaluated to assess their durability and structural integrity for oil–water separation applications. The analysis focused on their resistance to mechanical stress and flexibility. Tensile tests were repeated three times for each membrane sample, using measurements taken from different locations on the same specimen. Figure 7A displays the stress-strain curves of all membrane samples. The tensile strength of pristine PSU membrane was measured as 0.95 MPa, while the incorporation of SPTES nanoparticles into the membrane structure led to a 33.7% and 68.4% increase in tensile strength for the PSU@SPTES-1 and PSU@SPTES-3 samples, respectively. However, as the SPTES content was increased to 5, 7, and 10 wt %, the tensile strength declined by extent of 5.3, 17.9, and 46.3%, respectively. Obaid et al., investigated the mechanical behavior of neat PSU and PSU fibers containing 1 wt % SiO_2_.^20^ The tensile strength and strain values for neat PSU were reported as approximately 0.75 MPa and 23%, respectively, while those for PSU with 1 wt % SiO_2_ were around 0.70 MPa and 13%. In comparison, the pristine PSU nanofibers fabricated in this study exhibited a higher tensile strength of 0.95 MPa but a significantly lower elongation at break (∼7%). Similarly, PSU@SPTES-1 nanofibers showed an even higher tensile strength of 1.25 MPa with an elongation of 8%.

**Figure 7:**
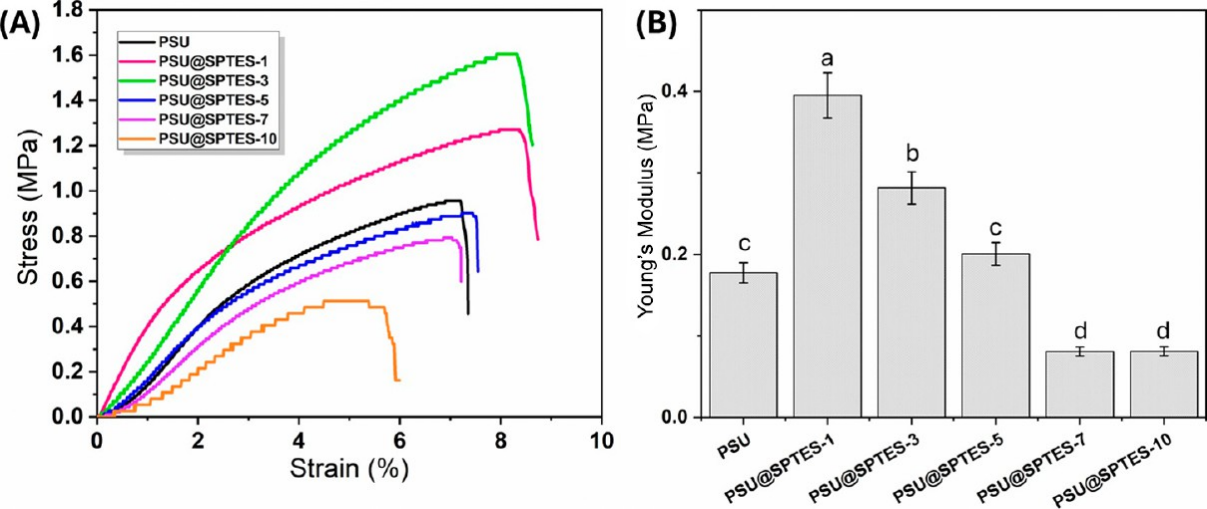
(A) Stress-strain curves, (B) Young’s modulus for the PSU membranes loaded with different SPTES nanoparticle concentrations.

It is noteworthy that in the reference study,^20^ the AFDs were approximately 600 nm for pristine PSU and 800 nm for PSU/SiO_2_ composites, whereas the fiber diameters in this study were significantly much smaller, measured as 107 ± 20 nm for pristine PSU and 147 ± 38 nm for PSU@SPTES-1. This difference in mechanical performance can be attributed to the substantial decrease in fiber diameter. The smaller fiber diameters likely led to a more interconnected and compact network, which enhanced the tensile strength but compromised the flexibility. This denser structure restricted the deformation ability of the fibers, and thus resulting in more brittle behavior. The reason for the considerable decrease in AFD for the PSU@SPTES-10 sample can be explained by the higher additive content, which induced the agglomeration of nanoparticles, and hence degraded the membrane structure, as reported in a previous study (Table 3).^20^ In other words, it can be concluded that the mechanical properties are associated with the AFD values of the membranes, as the PSU@SPTES-10 membrane exhibited the poorest performance.^42^

**Table 2:** SFO (Light Oil) Permeability and Separation Efficiency of PSU@SPTES-5

sample	oil flow rate (mL/h)	separation efficiency (%)
gravity-driven	91	86.5
pressure-driven	2709	98.6

**Table 3:** Comparison between the Oil Fluxes and Separation Efficiencies of Different Nanofiber Membranes

membrane type	type of oil	flux (L/m^2^ h)	separation efficiency (%)	ref
PAN-FPU/PSF	diesel	4024	99.6	^63^
PSF	diesel	5434	92.5	^63^
F/Cu/PDA/CF	CCl_4_	2924	99.5	^14^
F/Cu/PDA/CF	petroleum ether	3100	-	^14^
PVDF-SiO2	petroleum ether	3200	99.3	^7^
PAN/PS/FS-1.5 (forced)	Canola oil	613	-	^27^
bio-PU/TiCP2	*n*-hexane	4010	99.78	^64^
PSU@SPTES-5	Diesel	1479	99.4	this study
PSU@SPTES-5	CCl_4_	8211	96.6	this study
PSU@SPTES-5	petroleum spirit	11,722	99.9	this study
PSU@SPTES-5 (gravitational)	sunflower oil	91	86.5	this study
PSU@SPTES-5 (forced)	sunflower oil	2709	98.6	this study

The deterioration in tensile strength was negligible for PSU@SPTES-5, whereas it was substantial for the PSU@SPTES-7 and severe for PSU@SPTES-10. Moreover, the elongation at break exhibited a rising trend with SPTES loading until reaching 10 wt %. Compared to the pristine PSU membrane, the elongation at break improved by 16.3, 15.4, 3.3, and 0.1% for the PSU@SPTES-1, PSU@SPTES-3, PSU@SPTES-5, and PSU@SPTES-7, respectively. In contrast, PSU@SPTES-10 sample exhibited a 25.3% decrease in elongation at break relative to the neat PSU. This improvement in mechanical properties of the samples, except for PSU@SPTES-10, stem from the possible hydrogen bonding between residual silanol groups of hydrolyzed SPTES nanoparticles and the oxygen or sulfone groups of PSU.^43–45^ Beyond the fact that hydrogen bonding is the main interaction pathway, phenyl groups are also a major contributor to the interfacial interactions, albeit indirectly.^43,44^ Phenyl moieties enable cation−π or π–π stacking interactions with aromatics in PSU, and thus facilitating and stabilizing hydrogen bonding networks.^43,44^ When the membrane sample PSU@SPTES-5 was evaluated, the addition of SPTES nanoparticles at 5 wt % promoted a slight improvement in strain performance compared to pristine PSU and membrane samples with 1 and 3 wt % additive content. Nevertheless, in terms of tensile strength, PSU@SPTES-5 was insufficient to attain the level achieved by the sample with 3 wt % SPTES. Overall, 5 wt % SPTES-modified nanofiber membrane demonstrated an excellent performance in terms of strength and elongation, enhancing durability while maintaining adequate flexibility.

Young’s modulus was calculated for each membrane sample, as illustrated in Figure 7B. Young’s modulus reflects the stiffness of the nanofibrous membrane and its resistance to elastic deformation under applied stress.^46^ In this study, the incorporation of 1 wt % SPTES enhanced stiffness, indicating stronger fiber formation and a denser pore structure with closely packed fibers and fewer voids between them. The highest Young’ s modulus value (0.39 MPa) was recorded for the PSU@SPTES-1 sample. However, as the SPTES content increased to 3 wt % and beyond, a substantial decrease in modulus was observed. Particularly, the declines in Young’s modulus were 28.2%, 48.7%, 79.3%, and 79.2% for PSU@SPTES-3, PSU@SPTES-5, PSU@SPTES-7, and PSU@SPTES-10, respectively. The observed reduction originates from the deterioration in nanoparticle dispersion and agglomeration, which compromise fiber continuity at higher filler loadings by disrupting interfiber bonding and structural integrity. This trend aligns with previous observations in nanocomposite systems, where excessive filler concentration leads to agglomeration and a reduction in reinforcement efficiency.^20,42,47^ According to Tukey’s multiple comparison test, a statistically significant difference (*p* < 0.05) was observed between the Young’s modulus values of the additive-incorporated samples and the pristine PSU membrane, demonstrating the role of nanoparticles in enhancing mechanical performance.

In nanocomposites, the mechanical performance correlates with the degree of uniform nanoparticle distribution.^48^ The highest mechanical properties were obtained in the PSU@SPTES-1 and PSU@SPTES-3 samples, by virtue of uniform distribution of nanoparticles. However, higher SPTES loadings caused aggregation and resulted in lower modulus and tensile strength. This behavior is in accordance with the literature, which states that excessive filler amounts diminish the efficiency of the reinforcements.^49^

### 3.5 Thermal Stability

The effect of SPTES loading on the degradation behavior of the nanocomposite fibrous membranes was studied by recording TGA thermograms under nitrogen atmosphere, illustrated in Figure 8. Thermograms of the neat PSU and PSU@SPTES-5 nanofiber membranes exhibit a sharp weight loss between 437 and 600 °C corresponding to the main degradation of the polymer backbone. The initial decomposition temperature, 437 °C, of pristine PSU was enhanced to approximately 461 °C, upon the addition of SPTES nanoparticles into membrane matrix. The char yields at 800 °C are 30.9% and 12.2% for PSU@SPTES-5 and pristine PSU, respectively. It can be concluded that incorporation of nanoparticles into the nanofiber polymer matrix fosters the thermal stability by promoting the formation of a more stable network.^7,50^

**Figure 8:**
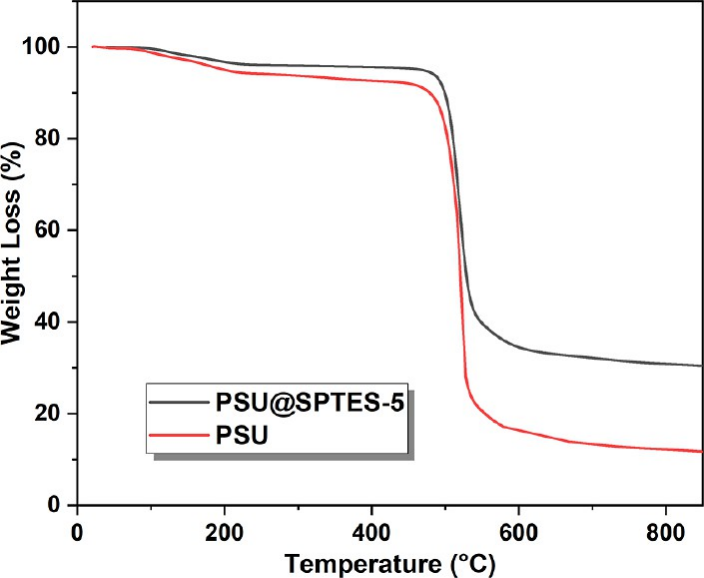
TGA thermograms of nanofiber membrane samples.

### 3.6 Oil–Water Separation Performance

Oil flux and separation efficiency tests for gravity-driven separation were conducted for all produced membranes, as demonstrated in Figure 9A. Diesel was chosen as the test oil to evaluate the oil–water separation performance of PSU nanofiber membranes prepared with varying SPTES additive ratios. Among the PSU membranes including 0, 1, 3, 5, 7, and 10 wt % SPTES, the specimen with 5 wt % SPTES (PSU@SPTES-5) exhibited the highest flux and separation efficiency, determined as 1479 L/m^2^h and 99.4%, respectively. Tukey’s multiple comparisons test demonstrated that PSU@SPTES-5 exhibited the highest flux and, together with all other SPTES-modified membranes, showed significantly better separation efficiency than pristine PSU (*p* < 0.05), underscoring its superior overall performance. Based on all these results, PSU@SPTES-5 membrane specimen was selected to investigate for further studies.

**Figure 9:**
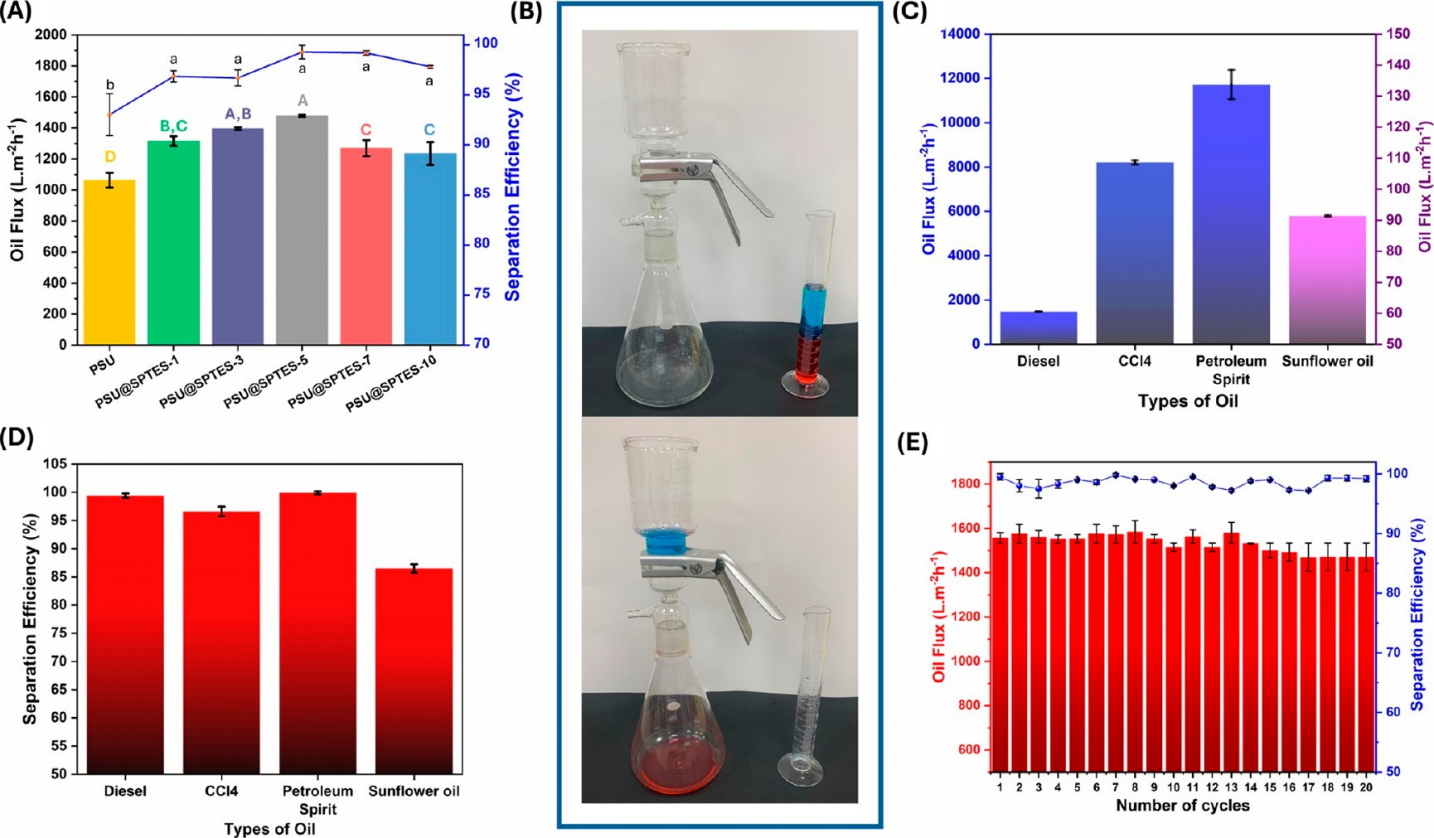
(A) Separation efficiency and oil flux of modified membranes, (B) experimental setup of PSU@SPTES-5 gravimetric separation test, (C) oil flux of PSU@SPTES-5 with different oil–water mixtures, (D) separation efficiency of PSU@SPTES-5 with different oil–water mixtures, and (E) cycle test of PSU@SPTES-5 with diesel oil.

The incorporation of SPTES nanoparticles into membrane structure resulted in an enhancement not only in oil flux and separation efficiency, but also in the mechanical properties, as discussed in the previous sections. The SPTES amount up to 3 wt % promoted an improvement in tensile strength, whereas filler contents higher than 3 wt % had an adverse effect on tensile strength. However, although a decrease was observed in PSU@SPTES-5 sample, it was negligible enough to be disregarded. Additionally, upon examining Young’s modulus, the highest value was observed for PSU@SPTES-1 sample. Despite exhibiting a 28.6% lower modulus than PSU@SPTES-3, the PSU@SPTES-5 membrane still retained an 11.1% higher modulus relative to pristine PSU. Similarly, while the elongation at break of PSU@SPTES-5 was 10.1% lower than that of PSU@SPTES-3, it was nearly identical to that of pristine PSU. The lower modulus of PSU@SPTES-5 may arise from a slight attenuation of interfacial bonding or early stage agglomeration. Nevertheless, maintaining a higher modulus with respect to pristine PSU revealed that the nanoparticle fraction in PSU@SPTES-5 sample influenced the mobility of the polymer chains to a negligible degree, maintaining the structural flexibility and preventing the embrittlement. In other words, PSU@SPTES-5 sample preserved an appropriate trade-off between stiffness and flexibility. Therefore, these characteristics endowed the membrane with improved durability under operational conditions.

The fundamental factors dictating the oil–water separation efficiency in nanofiber membranes are wetting state, multiscale roughness, pore morphology, and mechanical behavior.^51–54^ From the perspective of surface wettability, superhydrophobic nanofiber membranes with hierarchical surface roughness confer excellent flux and separation efficiency. Additionally, Young’s modulus has a greater influence on separation efficiency, since stiffness determines the stability of pore structure and resistance to compaction under pressure.^51^ As discussed above, the integration of SPTES nanoparticles into the PSU nanofiber structure enhanced the mechanical properties, which resulted in high separation efficiency consistent with the literature.^51^ PSU@SPTES-5, chosen as the sample with the optimal nanoparticle fraction, demonstrated the highest oil flux and separation efficiency with features of superhydrophobicity, high mechanical properties, long-term durability, and excellent chemical and UV stability.

Both gravity-driven and pressure-driven separation methods were applied to assess the membrane performance of PSU@SPTES-5. The gravity-driven separation was studied using four different oil–water mixtures: diesel, carbon tetrachloride (CCl_4_) (Video S2), petroleum spirit, and SFO through conducting experiments in triplicate for each type of oil to ensure reproducibility, shown in Figure 9C and D. Prior to each test, the membrane was prewetted with the corresponding oil to improve its selective wettability. In gravity-driven experiments, 40 mL of the oil–water mixture was poured into the separation setup. The PSU@SPTES-5 membrane allowed the oil to pass through quickly and be collected in the conical flask below, while water was effectively retained above the membrane due to its strong hydrophobicity and oleophilicity (Figure 9B). As shown in Figure 9C, the flux values were 1479 L/m^2^h for diesel, 8211 L/m^2^h for CCl_4_, 11,722 L/m^2^h for petroleum spirit, and 91 L/m^2^h for SFO. In comparison to the flux measured with diesel, the fluxes of CCl_4_ and petroleum spirit through PSU@SPTES-5 nanofiber membrane specimen was nearly 5 and 8 times higher, respectively. However, the flux attained with SFO was approximately 16 times lower than that of diesel. Since the dynamic viscosity of SFO is much higher than that of diesel, slower passage of SFO through the membrane is a predictable outcome.

The separation efficiency of the PSU@SPTES-5 nanofiber membrane was characterized by using oil–water mixtures prepared separately. As shown in Figure 9D, the separation efficiencies were measured as 99.4%, 96.6%, 99.9%, and 86.5%, for diesel, CCl_4_, petroleum spirit, and SFO, respectively. Among the tested oils, the highest separation efficiency of 99.9% was obtained with petroleum spirit, which can easily permeate through the membrane owing to its relatively low viscosity and low molecular weight. On the other hand, SFO demonstrated the lowest separation efficiency as 86.5%, since it has the highest viscosity and molecular weight compared to other test oils. Tong et al.^55^ examined whether membranes developed for organic solvent nanofiltration (OSN) tend to be hydrophilic or hydrophobic. They stated that polar solvents preferred hydrophilic OSN membranes to permeate, while hydrophobic OSN membranes facilitated the passage of nonpolar solvents. In accordance with this, the highest separation efficiency was attained with petroleum ether since it is the most nonpolar solvent among the others. Considering the dielectric constants of the test oils at 20 °C, they can be ranked in the descending order as follows: SFO > diesel > CCl_4_ > petroleum spirit.^56–59^ The solvent with the lowest dielectric constant has the highest nonpolarity, since there is an opposite relationship between the dielectric constant and nonpolarity.^55^ Accordingly, solvents with low dielectric constants exhibit the highest affinity toward hydrophobic membranes. The superhydrophobic PSU@SPTES-5 nanofiber membrane with a 154.3° WCA gave the highest separation efficiency of 99.9% with petroleum spirit, which possesses the lowest dielectric constant among the tested oils.

The durability of the PSU@SPTES-5 nanofiber membrane was investigated through repeated oil–water separation experiments using a diesel-water mixture, as illustrated in Figure 9E. After 20 separation cycles, membrane maintained a separation efficiency above 97.2% in all cases, while the permeate oil flux decreased from 1556 to 1470 L/m^2^ h, corresponding to a flux loss rate of approximately 5.5%. Throughout all cycles, the membrane consistently showed high separation efficiency and stable flux, highlighting its effectiveness for multiple uses in oil–water separation applications.

In order to test the pressure-driven separation performance of PSU@SPTES-5 membrane, setup shown in Figure 10 was utilized. A total of 20 mL of an oil–water mixture, including equal volumes of water and SFO was loaded into a syringe and pumped through the membrane at a flow rate of 2 mL/min. In the forced system, the time required for the oil phase to completely pass through the membrane was recorded. The oil was stained with Sudan IV dye, while the water was colored with methylene blue to visually distinguish the two phases. The oil–water separation experiment was repeated three times, and the oil permeability of the membrane was evaluated accordingly. At this point, the surface hydrophobicity of the PSU@SPTES-5 membrane generated a repulsive force against the pumped oil–water mixture, thus facilitating the selective passage of oil molecules through the membrane. The oil–water separation performance of the membrane was compared under gravity-driven and pressure-driven systems using SFO, as presented in Table 2. In the gravity-driven test, separation efficiency was measured as 86.5%, whereas it increased to 98.6% when a forced system was employed.

**Figure 10:**
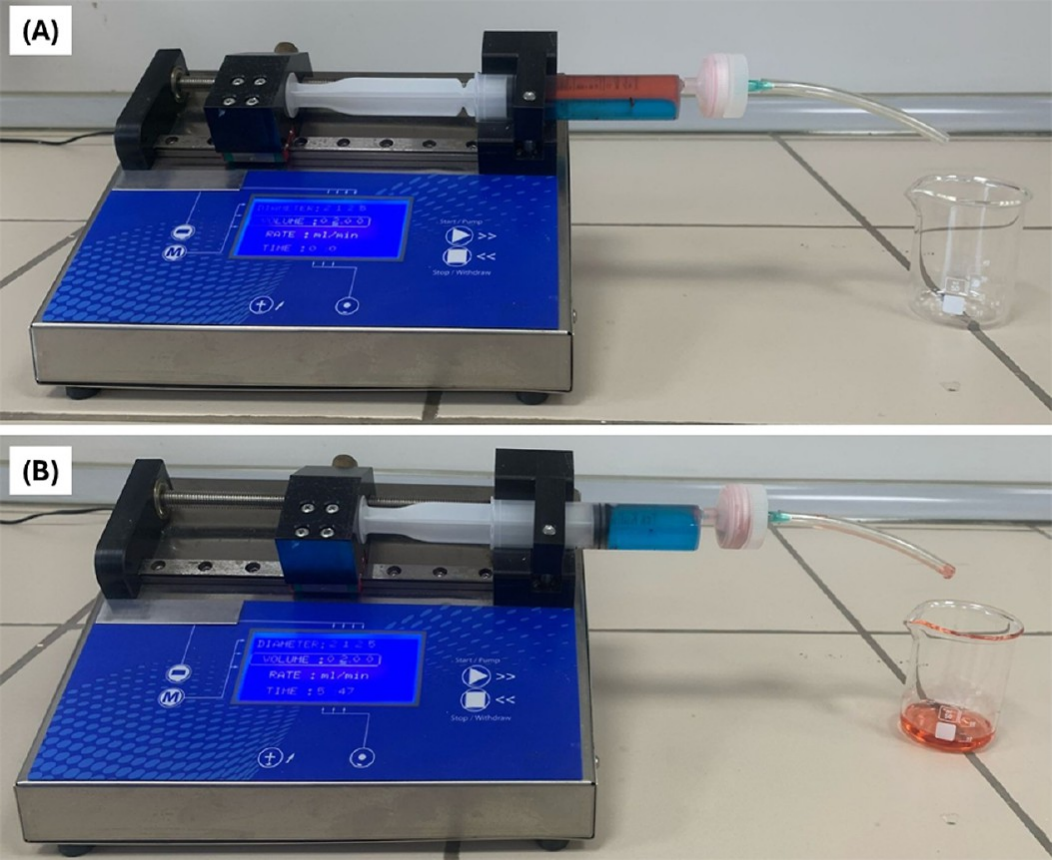
Forced separation setup using a syringe pump, (A) before, (B) after oil–water separation.

To further elaborate the performance of PSU@SPTES-5 sample, diesel and SFO-based emulsions were employed as representative modelsof oily wastewater. As illustrated in Figure 11, the feed emulsions contained abundant microsized water droplets, whereas the permeates appeared optically transparent, demonstrating the membrane’s excellent selectivity and efficiency for W/O emulsion separation.

**Figure 11:**
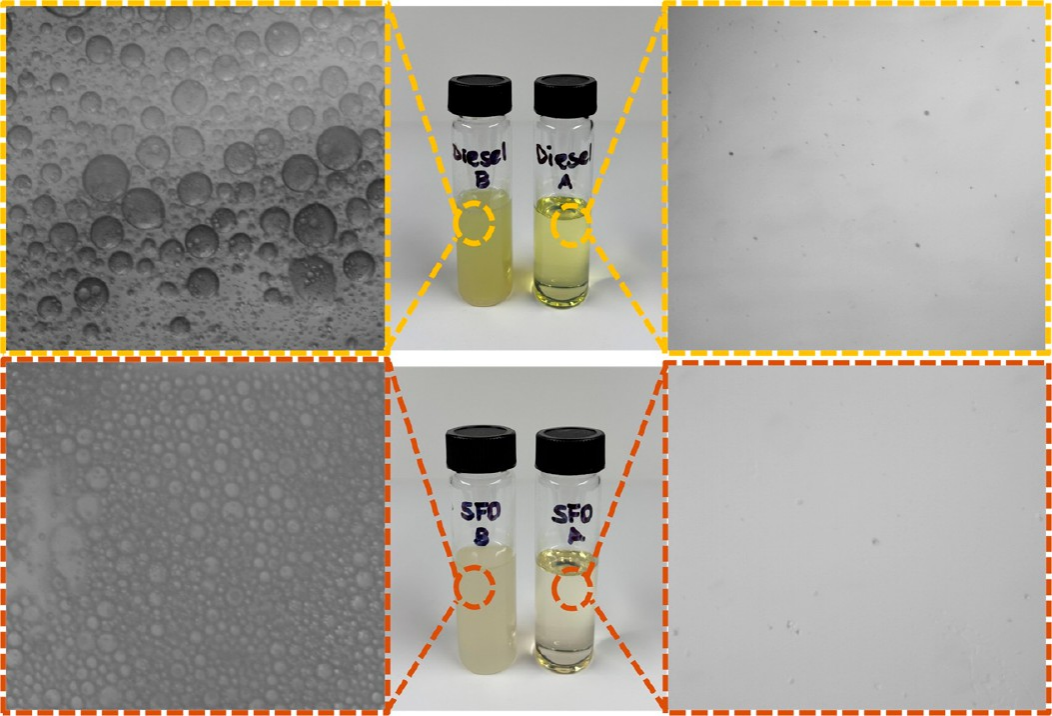
Photographs and optical images of diesel (top) and SFO (bottom) emulsions before and after permeation through PSU@SPTES-5.

When tested with emulsions prepared from diesel and SFO (99:1 = O/W (w/w), stabilized with 0.1 wt % Span 80), PSU@SPTES-5 showed a high separation efficiency and excellent flux when compared to the results obtained in the initial cycle tests of the as-prepared membrane. In the case of diesel emulsion, the membrane achieved a separation efficiency of 99.3% with a flux of 1203L/m^2^ h, which is comparable to the performance obtained under the nonemulsion condition (99.4% and 1479 L/m^2^ h, respectively). This slight reduction in flux can be attributed to the additional transport resistance caused by dispersed water droplets, consistent with general fouling mechanisms where foulant accumulation increases hydraulic resistance and reduces permeate flux.^60,61^ As supported by recent studies demonstrating that water accumulation in W/O systems can form a barrier layer that restricts flux without compromising separation efficiency.^62^ For the SFO emulsion, PSU@SPTES-5 reached a separation efficiency of 97.2 % with a flux of 638 L/m^2^ h, representing a marked improvement in efficiency compared to the nonemulsion case (86.5%) but a significant decline in flux (91 L/m^2^ h → 63 L/m^2^ h). These results clearly demonstrate that while emulsion feed conditions introduce additional hydraulic resistance that limits flux, they also facilitate enhanced retention of water droplets, thereby improving the overall separation efficiency, particularly for high-viscosity oils such as SFO.^60^

The stability of PSU@SPTES-5 membrane was systematically evaluated after exposure to acidic and alkaline media and direct sunlight (Figure 12A–F). Immersion in 2 M NaOH and 2 M HCl solutions for up to 24 h resulted in only a slight decrease in WCA, with values remaining above 150° (152.4° in NaOH and 151.9° in HCl). This negligible change demonstrates that the membranes retained their superhydrophobic nature, suggesting that both the surface chemistry and fiber morphology remained intact under corrosive conditions. Cycle tests with diesel oil further confirmed the strong chemical and environmental resistance of the membranes. The pristine sample showed an oil flux of 1470.9L/m^2^ h and a separation efficiency of 99.2% after 20 cycles. After immersion in 2 M HCl, the flux slightly decreased to 1449.1L/m^2^ h, while the separation efficiency remained high at 97.1%, whereas 2 M NaOH treated membranes maintained a flux of 1466.3L/m^2^ h with an efficiency of 99.3%. The membranes exposed to direct sunlight for 24 h exhibited the highest flux (1444.1 L/m^2^ h) with a separation efficiency of 97.1 %. All membranes sustained higher than 97% separation efficiency across 20 cycles, confirming their robustness. While minor variations in flux were observed depending on the treatment condition, these changes did not compromise the overall performance. These findings clearly demonstrate that PSU@SPTES-5 membrane exhibited excellent stability against acidic, alkaline, and UV stress, maintaining high separation efficiency and strong superhydrophobicity even after prolonged exposure times.

**Figure 12:**
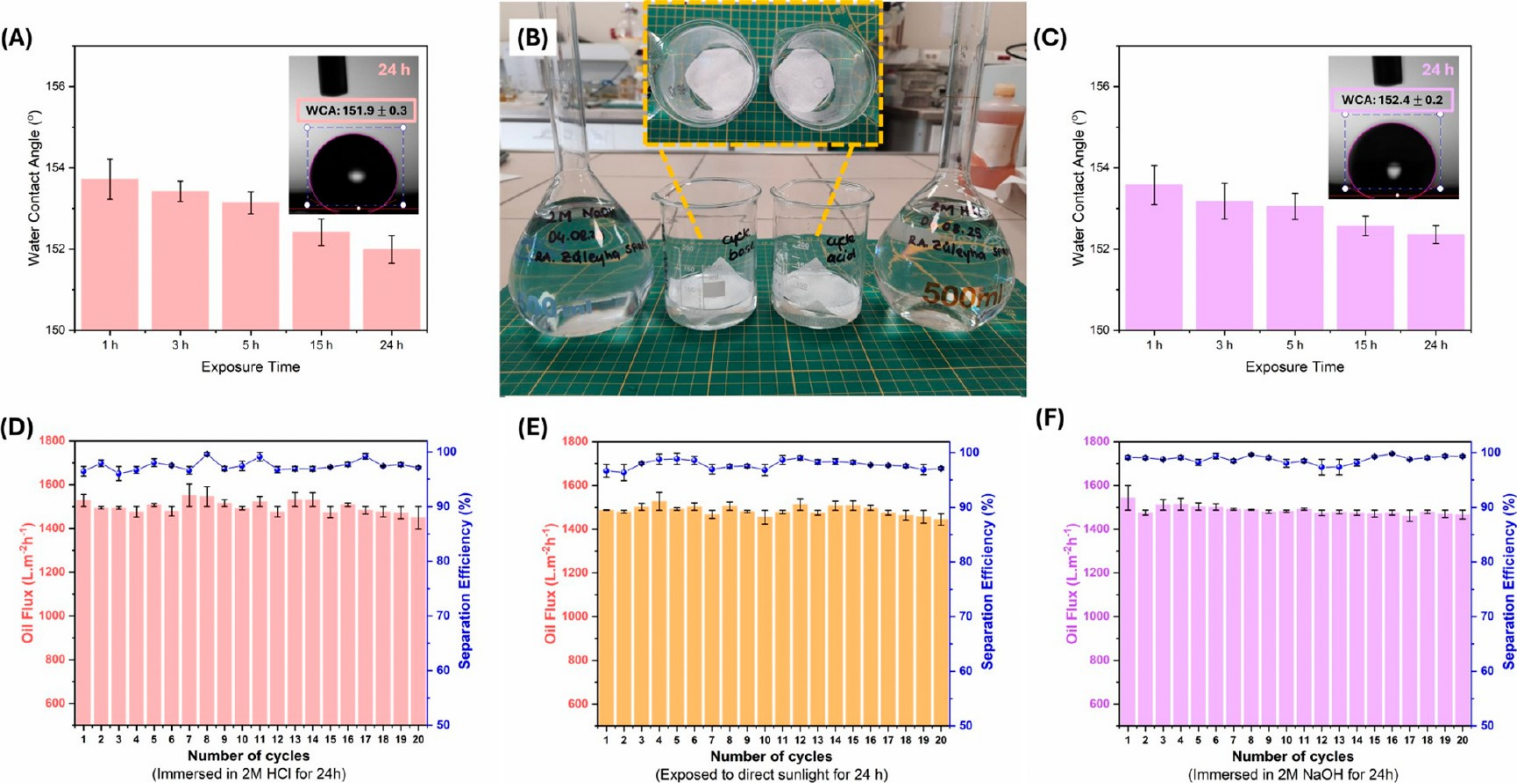
Stability of PSU@SPTES-5 membrane: (A) WCA change after immersion in 2 M HCI at different times, (B) visual appearance during acid/alkali immersion (24 h), (C) WCA change after immersion in 2 M NaOH at different times, (D–F) oil flux and separation efficiency over 20 diesel cycles after exposure to HCl, sunlight, and NaOH, respectively.

As seen from Table 3, PSU@SPTES-5 nanocomposite fibrous membrane, prepared for the first time in this study, exhibited high flux and separation efficiency along with superior membrane properties.

## 4 Conclusion

In this study, high flux, robust, and durable superhydrophobic nanocomposite fibrous membranes, which represent an advanced next-generation treatment, were developed for oil–water separation applications. For this purpose, superhydrophobic nanoparticles were prepared by the surface modification of nanoparticles with PTES. The sol–gel reaction was validated through FTIR and WCA measurements. Next, a series of electro-blown spun membrane samples were produced by the incorporation of varying amounts of SPTES nanoparticles into the PSU spinning solution. Upon investigating different additive loadings (ranging from 1–10 wt %), 5 wt % SPTES was determined to be the optimal amount, demonstrating the best properties in terms of membrane performance. After the incorporation of SPTES nanoparticles into the nanofibers, the WCA of PSU@SPTES-5 membrane sample was measured as 154.3° (within the superhydrophobic range), whereas the tensile strength decreased by only 5.3% and elongation at break improved by 3.3% in comparison to neat PSU membrane. However, tensile strength and elongation at break were enhanced in the samples with 1 and 3 wt % SPTES loading, which may result from the possible formation of hydrogen bonding between residual silanol groups of hydrolyzed SPTES nanoparticles and the oxygen or sulfone groups of PSU, accompanied by phenyl moieties that facilitate and stabilize the hydrogen-bonding networks. The presence of SPTES nanoparticles on the nanofiber network structure has also contributed to the improvement of thermal properties. When PSU@SPTES-5 was compared with pristine PSU sample, the initial decomposition temperature shifted from 437 to 461 °C, while char yield at 800 °C exhibited an increment from 12.2% to 30.9%. Furthermore, the oil flux of PSU@SPTES-5 membrane sample was measured as 1479 L/m^2^ h for diesel, 8211 L/m^2^ h for CCl_4_, 11,722 L/m^2^ h for petroleum spirit, and 91 L/m^2^ h for SFO. The membrane sample showed excellent durability and reusability, maintaining its separation efficiency higher than 97.2% after 20 cycles of repeated use without sacrificing its structural integrity. When tested using SFO, the vegetable oil with the highest dielectric constant and polar character, the separation efficiency of the membrane increased by nearly 14% when the system was switched from gravity-driven to pressure-driven mode. In the case of the emulsions prepared with diesel and SFO, separation efficiencies of 99.3% and 97.2% were obtained, respectively, for the PSU@SPTES-5 membrane sample. Even under harsh conditions such as 2 M HCl, 2 M NaOH, and UV irradiation for 24 h, the sample demonstrated a high separation efficiency after 20 cycles of repeated use. Thus, high-flux, robust, and durable superhydrophobic nanocomposite fibrous membranes produced via EBS, accompanied by the incorporation of SPTES nanoparticles, offer promising alternatives with excellent properties for oil–water separation applications.

## Supplementary Material





## Notes

A visual comparison of the PTES-modified sample before and after modification in the reaction R(0.6)—Video S1 (MP4)
The gravity-driven carbon tetrachloride (CCl_4_)-water separation—Video S2 (MP4)

## Abbreviations

TiO_2_: titanium dioxide
SiO_2_: silicon dioxide
and Fe_3_O_4_: iron­(II,III) oxide
NH_4_OH: ammonium hydroxide
C_2_H_5_OH: ethyl alcohol
W/O: water-in-oil
EBS: electro-blow spinning
PSU: polysulfone
PTES: phenyltriethoxysilane
SPTES: PTES-modified silica
WCA: water contact angle
PVDF: polyvinylidene fluoride
PVP: polyvinylpyrrolidone
PET: polyethylene terephthalate
PAN: polyacrylonitrile
GO: graphene oxide
PFOTES: perfluorooctyltriethoxysilane
DMF: *N*,*N*-dimethylformamide
IPA: isopropyl alcohol
CCl_4_: carbon tetrachloride
SFO: sunflower oil
R: the PTES/SiO_2_ weight ratio
FTIR: Fourier transform infrared spectroscopy
ATR: attenuated total reflectance
DLS: dynamic light scattering
SEM: scanning electron microscopy
AFD: the average fiber diameter
TGA: thermogravimetric analysis.
